# Case series of in situ pelvic floor reconstruction combining levator ani suture and negative pressure wound therapy for abdominoperineal resection

**DOI:** 10.1016/j.amsu.2019.05.014

**Published:** 2019-05-31

**Authors:** Eisaku Ito, Masashi Yoshida, Hironori Ohdaira, Masaki Kitajima, Yutaka Suzuki

**Affiliations:** Department of Surgery, International University of Health and Welfare Hospital, Japan

**Keywords:** Negative pressure wound therapy, Pelvic floor reconstruction, Abdominoperineal resection

## Abstract

**Background:**

Abdominoperineal resection (APR) is a standard surgical technique for low rectum cancer with a low recurrence rate. There are some problems associated with APR such as perineal hernia and perineal surgical site infection. Recently, the prophylactic efficacy of negative pressure wound therapy (NPWT) for surgical site infection has been reported. Herein, we analyzed the efficacy of in situ pelvic floor reconstruction combining levator ani suture and NPWT after APR for perineal hernia and perineal surgical site infection.

**Methods:**

We analyzed six patients treated by laparoscopic APR with NPWT combined with levator ani suture retrospectively. The primary endpoints were surgical site infection within 30 days and perineal hernia within 1 year after surgery. The day following surgery, we performed NPWT for the perineal wound using the VAC^®^ abdominal wound management system (KCI, San Antonio, TX, USA).

**Results:**

There were four male and two female patients ranging in age from 69 to 86 years (mean: 76 years). The mean NPTW duration was 17 days (13–20 days). The length of the postoperative hospital stay was 14–22 days (median: 18 days). There was no patient with surgical site infection within 30 days or with perineal hernia within 1 year after surgery.

**Conclusion:**

We experienced the in situ pelvic floor reconstruction combining levator ani suture and NPWT after laparoscopic APR for perineal hernia and perineal surgical site infection. This combination treatment was safe and may be effective for preventing surgical site infection and perineal hernia.

## Highlights

• Although APR has some unresolved problems such as perineal hernia and perineal surgical site infection.
• NPWT decreased perineal wound infection after APR but not reduced deep wound infection rates.
• Our procedure was able to reconstruct without foreign materials and provided sufficient separation from internal organs to perform safe NPWT.
• The in situ pelvic floor reconstruction combining levator ani suture and NPWT after laparoscopic APR was safe and may be effective for preventing surgical site infection and perineal hernia.

## 1 Introduction

Abdominoperineal resection (APR) is a standard surgical technique for low rectum cancer with a low recurrence rate. There are some problems associated with APR such as perineal hernia and perineal surgical site infection [1,2]. Recently, the prophylactic efficacy of negative pressure wound therapy (NPWT) for surgical site infection has been reported [[3], [4], [5]]. Herein, we experienced the in situ pelvic floor reconstruction combining levator ani suture and NPWT after laparoscopic APR for perineal hernia and perineal surgical site infection.

## 2 Materials and methods

### 2.1 Patients and data collection

Between September 2015 and March 2016, eight patients underwent laparoscopic APR for low rectum carcinoma. All operations were performed by an experienced surgeon who was also a licensed attending doctor for laparoscopic surgery. The primary endpoints were surgical site infection within 30 days and perineal hernia within 1 year after surgery. The indication for the APR was advanced low rectum cancer out of indication for intersphincteric resection (ISR) [6,7]. This paper has been reported in line with the PROCESS criteria [8].

### 2.2 Laparoscopic procedure (Fig.1)

Under general anesthesia, patients were placed in the reverse Trendelenburg position with legs spread. After pneumoperitoneum was established using the open technique at the umbilicus, a flexible electrolaparoscope was introduced through the umbilical port. Four operating ports were placed in the lower abdomen. Four additional ports (5 mm or 12 mm in diameter) were placed in the lower abdominal area. After the left-side colon had been mobilized with extended lymphadenectomy, the exposed rectum was adequately mobilized at the height of the levator ani muscle.

**Fig. 1:**
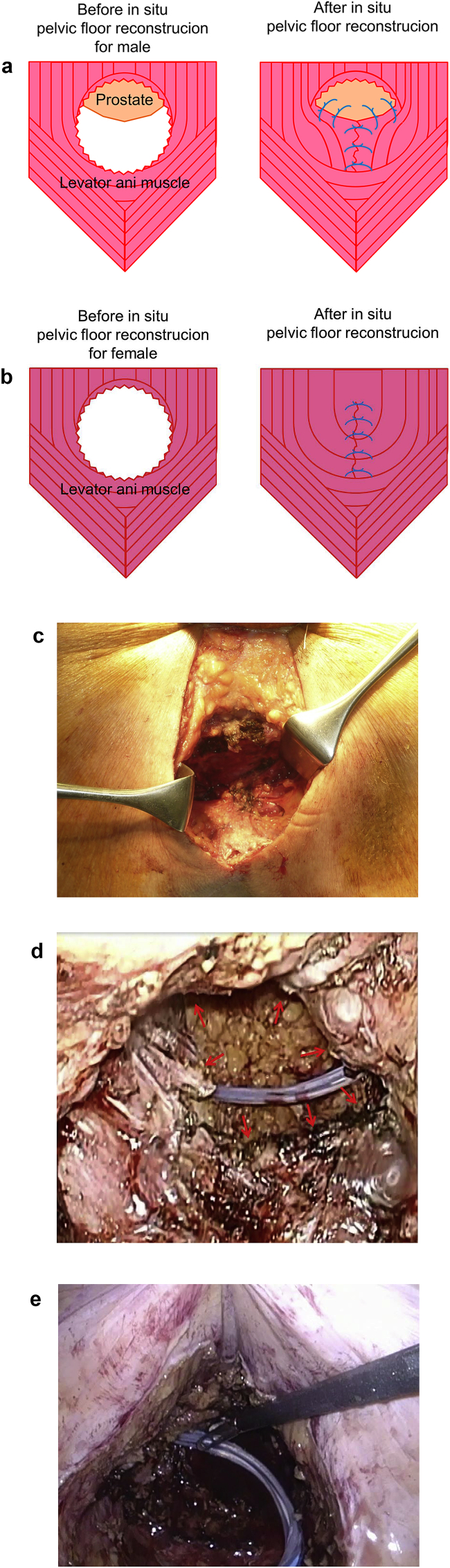
**a** The schema of the levator ani closure with prostate suture in a male. **b** The schema of the levator ani closure with prostate suture in a female. **c** The picture of the levator ani closure with prostate suture. **d** Laparoscopic view without the levator ani closure. The levator ani was not able to be sutured (arrow). **e** Laparoscopic view after the levator ani closure.

### 2.3 Perineal procedure

A spindle-shaped skin incision was made around the anus with a 3-cm margin. The rectum and perirectal fat tissue were excised in a lump. After deterging the wound and achieving hemostasis, we sutured the levator ani muscle using 0 monofilament absorbable threads. For female patients, the levator ani muscle was sutured with complete transfixion. Because the pelvic floor in a female is wider than a male, there is more residual levator ani muscle after rectal resection and easier suturing in a female. In contrast, for male patients, the levator ani muscle was sutured completely while suturing the prostate and ventral edge of the defect of the levator ani muscle. After suturing the prostate, we evaluated the mobility of the urinary catheter and judged whether to suture the urinary tract. We packed gauze in the pelvic cavity and completed the perineal procedure.

### 2.4 Stoma creation

After the perineal procedure, we placed a 19-Fr Blake drain tube at the pelvic surface of the sacrum. Finally, we induced the sigmoid colon extraperitoneally and closed the abdominal wound. We created a single-orifice sigmoidostomy using 3–0 monofilament absorbable threads.

### NPWT technique (Fig.2)

The day following surgery, we removed the perineum wound dressing and gauze and evaluated the wound bleeding and air leakage from the intra-abdominal drainage tube. We performed NPWT for the perineal wound using the VAC^®^ abdominal wound management system (KCI, San Antonio, TX, USA), unless there was wound bleeding. The setting was continuous vacuum, the suction pressure was 125 mmHg, and the dressing was changed every 48 h. When changing a sponge form, we sutured the subcutaneous cavity granulation tissue in a step-by-step manner and continued NPWT, unless there was wound infection. At the time of wound adaptation, we removing any previous sutures and added an additional stitch. These procedures were performed in a step-by-step manner until complete wound closure was achieved. After completing wound closure, we performed incisional NPWT 2 days later. The criteria for removal of the intra-abdominal drainage tube was daily discharge less than 30ml.

**Fig. 2:**
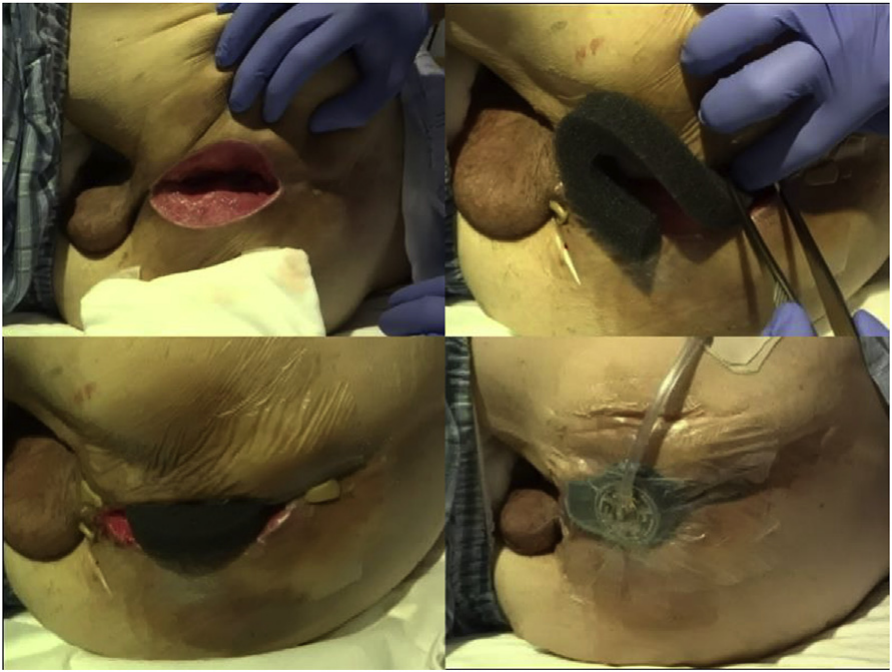
NPWT technique.

## 3 Results

### 3.1 Patient characteristics

In six of the eight patients who underwent laparoscopic APR the levator ani suture was successfully performed. The other two patients were not able to undergo the levator ani suture due to the fragility of their levator ani. Of these six patients, four were male and two were female, with an age range of 69–86 years (mean: 76 years). One patient underwent neo adjuvant chemo-radio therapy. There were four patients with an ASA score of 2 and two patients with a score of 3. On the other hand, the patient characteristics of the two patients whom we were unable to perform this technique was that; both male, with age of 66 and 77 years and ASA score of both cases were 3. No patient of eight underwent lateral lymph node dissection.

### 3.2 Surgical outcomes

**Fig. 3:**
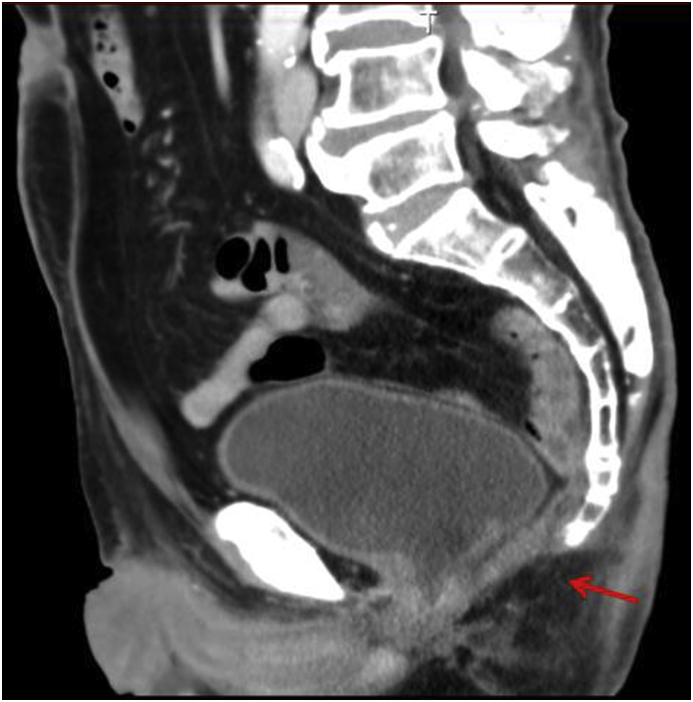
The post-operative CT scan showed that the levator ani was closed with prostate suture (arrow).

Surgical duration was 274–444 minutes (mean 379 minutes) and blood loss was 10–200 ml (mean 112 ml). All the patients were diagnosed with adenocarcinoma. The pathological stages were T2: one case, T3: five cases, N0: five cases and N3: one case. The intra-abdominal drainage tube was removed 5–19 days after the surgery (mean 18 days). The mean NPWT duration was 17 days (13–20 days). The length of the postoperative hospital stay was 14–22 days (median: 18 days). The postoperative complications included only a single case of bowel obstruction treated with conservative therapy. There was no patient with surgical site infection within 30 days or with perineal hernia within 1 year after surgery (Fig. 3).

## 4 Discussion

Herein, we evaluated the efficacy of the in situ pelvic floor reconstruction combining the levator ani suture and NPWT after laparoscopic APR for perineal hernia and perineal surgical site infection. This combination treatment was safe and effective for preventing surgical site infection and perineal hernia.

The treatment for rectal cancer is still challenging due to its high recurrence rate after surgery and the difficulty of the surgical technique. Since 1931, when Miles proposed APR for low rectal cancer, this procedure had been the standard treatment, leading to significant improvement in local recurrence rates [9]. However, extralevator APR (ELAPR) with complete resection of the pelvic floor muscles is superior to conventional APR (CAPR), decreasing local recurrence for advanced low rectal cancer without a substantial surgical margin [[10], [11], [12]]. ELAPR requires reconstruction using a skin flap and is invasive and carries a high risk for wound infection. Although lateral invasive advanced rectal cancer requires ELAPR, advanced low rectal cancer without lateral invasion is a good candidate for CAPR. Of course, there are some problems associated with APR such as perineal hernia and wound infection.

Although incisional NPWT decreased perineal wound infection after APR, incisional NPWT is not able to reduce deep wound infection rates [[3], [4], [5]]. NPWT for the pelvic cavity carries a risk of abdominal organ injury. Therefore, it is important to provide sufficient separation from internal organs to perform safe NPWT [13].According to past reports, pelvic floor reconstruction using skin flap and mesh implantation is effective for separating the abdominal and pelvic cavities [[14], [15], [16], [17], [18]]. In our method, these reconstruction techniques are unnecessary due to the suturing of the levator ani muscle. In cases where suturing the levator ani muscle was impossible, we sutured the patient's prostate without any prostate-related complications. Before suturing the levator ani and prostate, it is important to exclude prostate disease using CT scan. Moreover, we determined complete separation of the abdominal and pelvic cavities based on air leakage measurements from the intra-abdominal drain tube with negative pressure. Since there is a risk of abdominal organ injury using NPWT with incomplete pelvic cavity separation, closing the in situ pelvic floor by suturing the levator ani muscle played an important role in maintaining the safety of NPWT and avoiding perineal wound infection and perineal hernia.

We experienced the in situ pelvic floor reconstruction combining the levator ani suture and NPWT after laparoscopic APR for perineal hernia and perineal surgical site infection. This combination treatment was safe and may be effective for preventing surgical site infection and perineal hernia.

## Ethical approval

This study was reviewed by the Institutional Review Board of the International University of Health and Welfare Hospital and informed consent was waved due to the retrospective nature of this study (13-B-340).

## Funding

None.

## Author contribution

EI: study desing, data collections, data analysis, writing.

MY: data collections, critical Revision.

HO: data collections, critical Revision.

MK: critical Revision.

YS: critical Revision.

## Conflicts of interest

None.

## Research registration number

UMIN000036322.

## Guarantor

Eisaku Ito is a guarantor of this article.

## Provenance and peer review

Not commissioned, externally peer reviewed.

## Appendix A Supplementary data

Process Checklist 2018 Process Checklist 2018
